# Clock genes, hair growth and aging

**DOI:** 10.18632/aging.100130

**Published:** 2010-03-18

**Authors:** Mikhail Geyfman, Bogi Andersen

**Affiliations:** Departments of Medicine and Biological Chemistry, University of California Irvine, Irvine CA 92697, USA

**Keywords:** Circadian clock, cell cycle, hair growth cycle, aging, hair loss

## Abstract

Hair follicles undergo continuous cycles of growth, involution and rest. This process, referred to as the hair growth cycle, has a periodicity of weeks to months.  At the same time, skin and hair follicles harbor a functional circadian clock that regulates gene expression with a periodicity of approximately twenty four hours.  In our recent study we found that circadian clock genes play a role in regulation of the hair growth cycle during synchronized hair follicle cycling, uncovering an unexpected connection between these two timing systems within skin. This work, therefore, indicates a role for circadian clock genes in a cyclical process of much longer periodicity than twenty four hours.

## The hair growth cycle

Beginning after completion of hair morphogenesis (postnatal day 14 in the mouse), hair growth cycles commence with catagen, an involution process of the hair follicle during which the majority of its epithelial compartments undergo apoptosis [1].  This stage is followed by telogen during which the hair follicle remains in relative quiescence.  Telogen is interrupted by activation of epithelial stem and progenitor cells located in specialized stem cell compartments in the bulge and secondary hair germ, followed by rapid proliferation and differentiation of progeny keratinocytes; this growth phase is referred to as anagen [2].  In mice, the two initial hair growth cycles are synchronized such that the majority of hair follicles are in a similar stage of the hair growth cycle at a given time. But as the mouse ages, the hair growth cycles become progressively less synchronized [3].

## Circadian gene expression is hair growth cycle dependent

In order to systematically discover transcriptional activity associated with the hair growth cycle, we profiled mRNA expression at a genome-wide level over multiple time points corresponding to morphogenesis and two synchronized hair growth cycles. Interestingly, a large fraction of the genome, more than six thousand genes, exhibits changes in expression that correlates with the progression of the hair growth cycle, thus underscoring the complexity of this process [4,5].    One of the surprises that came from this study was the finding that genes regulated by the core circadian clock mechanism showed expression changes that correlated with the hair growth cycle, with highest expression during the telogen-anagen transition.

**Figure 1.**
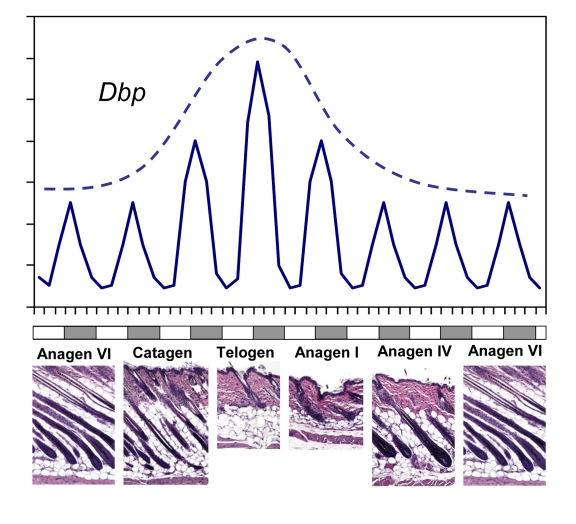
CLOCK-controlled gene expression in skin has a circadian pattern and correlates with synchronized hair growth cycles. A schematic diagram showing rhythmic circadian expression of clock controlled gene *Dbp* over different phases of the hair growth cycle (solid line).  The circadian amplitude of *Dbp *expression correlates with progression of the hair follicle cycle with highest expression during telogen (broken line). Skin histology for representative hair growth cycle stages is shown below. Note that this schematic does not show the actual length of each phase of the hair growth cycle.

On a molecular level, the circadian clock consists of positive and negative feedback loops.  At its core are the bHLH-PAS transcriptional activators CLOCK and BMAL1 (ARNTL), which form a heterodimer and activate target genes containing E-boxes in their enhancer regions, including Periods (Per1, 2 and 3) and Cryptochromes (Cry1 and 2).  PERs and CRYs form heterodimeric complexes that translocate into the nucleus where they inhibit BMAL1-CLOCK transcriptional activity, thus constituting the negative feedback loop [6]. In other words, the PER/CRY complex inhibits its own expression, allowing for reactivation of BMAL1/CLOCK leading to rhythmic expression with a periodicity of 24 hours. Several other proteins, including kinases, play an important role in generation of rhythmic expression [7]. The CLOCK-BMAL1 heterodimer activates other genes as well, including Dbp, Tef, Hlf, and Rev-Erbα, which codes for an orphan nuclear receptor.  REV-ERBα regulates transcription of Bmal1 and other target genes by binding to retinoic acid-related orphan receptor response elements (ROREs) [8].  The clock genes that we identified as upregulated in telogen/early anagen were all CLOCK/BMAL1 target genes, including Pers, Dbp and Rev-Erbα. While these genes show a clear circadian pattern of expression in skin, as was previously demonstrated [9-11], their amplitude was higher during telogen and early anagen (Figure 1), indicating that in skin, the expression of clock controlled genes is dependent both on circadian mechanism and the hair growth cycle.

**Figure 2.**
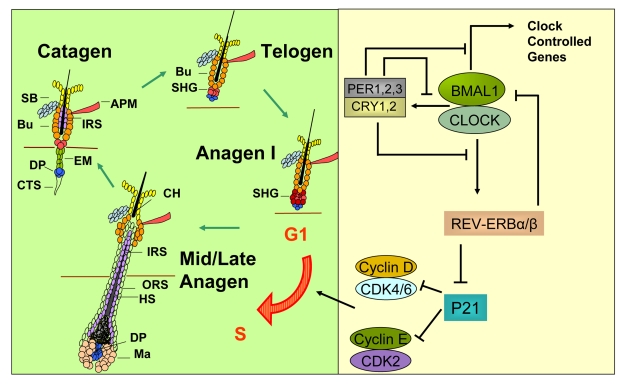
A model for how circadian clock genes participate in regulation of the synchronized hair growth cycle through regulation of cell cycle progression in the secondary hair germ. The hair growth cycle (left panel) is a continuous process consisting of the quiescent telogen phase followed by the growth phase (anagen) where signals, presumably originating in the dermal papilla, activate stem and progenitor cell proliferation leading to growth and differentiation of the hair shaft. Anagen is followed by catagen where the lower two-thirds of the follicle undergo apoptosis, sparing the stem cell compartments and the dermal papilla.   The CLOCK/BMAL1 complex is at the core of the mammalian circadian clock mechanism (right panel). It activates multiple genes, collectively referred to as clock controlled genes.  Among these genes are *Per1,2,3* and *Cry1,2 whose *protein products translocate into the nucleus to inhibit the transcriptional activity of the CLOCK/BMAL1 complex.  *Rev-erbα* is another clock controlled gene whose protein product negatively regulates expression of *Bmal1*.  Additionally, REV-ERBα directly inhibits expression of the G1-S cell cycle inhibitor p21^WAF1/CIP^. In the absence of BMAL1, downregulation of *Rev-erbα* leads to high *P21* expression and G1 arrest in the hair germ cells during anagen I of the hair follicle cycle, thus delaying anagen progression. APM - arrector  pili muscle,  Bu - bulge, CH - club hair, CTS - connective tissue sheath, DP - dermal papilla, EM - epithelial membrane, HS - hair shaft, IRS - inner root sheath, Ma - matrix, ORS - outer root sheath, SB - sebaceous gland, SHG - secondary hair germ.

Since our mRNA expression studies were performed using whole skin, we asked which compartments of the skin and hair follicles contribute to the robust rhythmic circadian gene expression in telogen.  The hair follicle contains several functionally and structurally distinct compartments, including the bulge region, which harbors slow-cycling hair follicle stem cells [12]; the secondary hair germ, which contains actively cycling stem and progenitor cells [13-15]; and the dermal papilla, a source of signals for activating the stem cells at the beginning of anagen [16] (Figure 2).  While in situ hybridization studies revealed that all cell types of the skin express circadian clock genes, the site of most prominent rhythmic circadian gene expression during telogen and early anagen was the secondary hair germ.  This compartment, strategically positioned between the dermal papilla and the bulge, contains proliferative Lrg5-positive stem cells thought to have migrated from the bulge during late catagen and early telogen [13,17].  The secondary hair germ cells are the first to be activated during anagen initiation, giving rise to transient amplifying cells of the hair matrix and eventually differentiating into the hair shaft [15,17,18]. Additionally, our data shows that as anagen progresses, the circadian amplitude within the hair follicle proper becomes dampened, while the circadian amplitude in the dermis and interfollicular epidermis continues to be robust. Interestingly, suspension of circadian rhythm has been previously noted in other highly proliferative and differentiating tissues, including testis and thymus [19-21].

## Role for circadian clock genes in hair growth cycling

The robust circadian clock gene expression within the secondary hair germ led us to test the possibility that circadian clock genes might play a role in the hair growth cycle.  For these studies, we turned to Clock and Bmal1 mutant mouse models. We found a significant delay in anagen progression in both mutants and this delay was more pronounced in Bmal1 deficient mice, possibly due to partial functional redundancy between Clock and its homologue Npas2. Clock and Bmal1 mutant mice have no visible defects in hair follicle morphogenesis and enter the first stage of anagen, characterized by the expansion of the secondary hair germ, at approximately the same time (post-natal day 22).  Yet by day 28, when the majority of hair follicles in control  littermates have  developed hair matrix  and hair shaft with the hair bulb growing into the subcutis, the Bmal1 mutant mice remained in the first anagen phase [5].  After experiencing a nearly week-long delay, the Bmal1 deficient hair follicles resumed normal progression of the hair cycle.  There were no abnormalities in the structure of the mature anagen follicles in the Bmal1 or Clock mutant mice, supporting the idea that circadian clock genes are primarily involved in timing mechanisms during the telogen-anagen transition.

Further analysis revealed absence of mitotic cells in the early anagen secondary hair germ in Bmal1 mutant hair follicles, while wild-type secondary hair germs at the same stage contained mitotic cells. Importantly, epidermis and dermis of Bmal1 mutant mice contained mitotic cells, indicating that the proliferation defect was hair follicle specific. Phosphorylated Retinoblastoma Protein (Rb), a marker of cell cycle progression through the G1-S cell cycle checkpoint [22], was absent  in the secondary hair germ of Bmal1 mutant hair follicles while it was abundant in control mice. These results indicate that in Bmal1 mutant hair follicles, progenitor cells of the early anagen secondary hair germ are arrested at the G1-S cell cycle checkpoint.

To gain insights into the molecular mechanisms underlying the G1 arrest of progenitor cells in the secondary hair germ, we profiled gene expression in the skin of Bmal1 deficient mice during telogen.  As expected, the expression of multiple known CLOCK-BMAL1 target genes was affected, including that of Rev-Erbα, which was downregulated approximately fifteen fold.  Studies in hepatocytes have demonstrated that REV-ERBα directly represses expression of the gene encoding the G1 cell cycle inhibitor p21WAF1/CIP [23], and consistently p21 is upregulated approximately 2.5 fold in Bmal1 mutant skin.   These findings led us to propose that hair growth cycling in Bmal1 mutant mice is delayed due to upregulation of p21, leading to slowed G1-S cell cycle progression in progenitor cells of the secondary hair germ (Figure 2).  These results are consistent with the known extensive crosstalk between the circadian clock and the cell division cycle [24].  We have also considered the possibility that circadian gene regulation of the hair growth might involve a mechanism that "counts" the number of circadian peaks to regulate timing in the hair growth cycle. However, results from our preliminary experiments in mice entrained to 22 and 26 hour days argue against this possibility.

In the mouse, the first two hair growth cycles are synchronized.  After completion of the second telogen, which can last up to thirty days, the coat begins to grow asynchronously in complex domains created by waves of anagen moving through the domain until a wave reaches "refractory" telogen, an area of skin unresponsive to the propagating anagen stimulus.  As the mouse ages, this process creates increasingly complex patterns of hair growth with each domain consisting of a telogen competent to be activated, a propagating anagen wave, a catagen, and a refractive telogen [3,25].  In preliminary experiments, we did not observe differential expression of clock controlled genes in skin corresponding to different hair growth phases in asynchronously cycling skin, suggesting the possibility that hair cycle related regulation of clock gene expression may be particularly important in synchronized hair follicle cycling.

One plausible role for circadian mechanisms in the hair growth cycle is in animals with seasonal hair growth, commonly found in mammals living in the wild [26-30]. In these animals, the circadian clock-regulated hormones melatonin and prolactin are thought to be key regulators of seasonal changes in hair growth [31-34]. Intriguingly, seasonal hair growth has been found to be regulated at the telogen-anagen transition; in several breeds of sheep, the winter coat is in telogen and in the spring when duration of daylight increases, hair follicles enter anagen and the winter hair fibers are shed [35].  Therefore telogen, and specifically the secondary hair germ, could serve as an important interpreter of photic hair growth cycle timing in animals bearing seasonal fur.

## The aging hair follicle

Hair loss and hair graying are commonly recognized symptoms of aging in mammals. In addition to the visible location of hair, the highly regenerative nature of hair follicles may explain why hair loss is a prominent feature of aging syndromes. Several mouse models with premature aging phenotypes show progressive hair loss or graying [36-38], and human conditions with progeria-like symptoms, such as Werner Syndrome and Hutchinson-Gilford Progeria, present with premature hair loss or hair graying [39]. The common form of human hair loss, androgenetic alopecia, shows a clear age-related progression [40]. In addition, some authorities have argued for a distinct age-related entity, referred to as senescent alopecia [40-43]. This syndrome as well as androgenetic alopecia are characterized by a reduction in the large diameter pigmented (terminal) hair and an increased prevalence of thin (vellus-like) hair [40].  Thus, both syndromes are thought to represent hair growth cycle defects characterized by increased telogen to anagen hair follicle ratio due to a shortened anagen phase and persistent telogen follicles [42,44].

Age-associated hair graying has been linked to ultraviolet light and reactive oxygen species (ROS)-induced cell damage.  The hair follicle bulge harbors melanocyte stem cells that give rise to mature melanocytes which synthesize and secrete hair pigments during anagen.  Graying human hair follicles have been shown to contain melanocytes with accumulated oxidative stress [45], and consistent with this finding, genotoxic stress induced by ionizing radiation in mice leads to premature differentiation of melanocyte stem cells, followed by stem cell depletion and hair graying [46].  Furthermore, deficiency in the ATM gene enhanced ectopic differentiation of the melanocyte stem cells [46]. Additionally, both Werner Syndrome and Hutchinson-Gilford Progeria are associated with accumulation of DNA damage [47].  Together, this data suggests that hair graying may be due to melanocyte stem cell depletion caused by UV radiation and genotoxic ROS.

The mechanisms underlying senescent alopecia have not been extensively studied. However, a plausible hypothesis is that analogous to hair graying, alopecia is related to loss of hair follicle epithelial stem cells either through decreased renewal, premature differentiation, apoptosis or cellular senescence. While the link between genotoxic stress and hair graying has been established using mouse models, only correlative data from human patients are available in regards to life-long UV exposure and hair loss [40,45].  Skin and hair follicles are heavily bombarded by UV radiation and also contain actively dividing keratinocytes, a likely source of mito-chondrial ROS.

## Circadian clock and the aging hair follicle

Recent studies indicate that circadian clock proteins may be involved in DNA repair and in regulating accumulation of cellular ROS, thus making them plausible actors in the aging processes [48].  Fu et. al. demonstrated that a mutation in the Per2 gene leads to an increase in tumor development as well as hair graying and hair loss after gamma irradiation [49]. PER1 is also known to interact with ATM and CHK2, thus affecting the proper initiation of double strand break repair [50].  In addition, recent work from several groups has revealed an important link between the circadian clock and cellular metabolism [51-54].  This work suggests that cell division and DNA synthesis are temporally segregated from the oxidative phase of the metabolic cycle [55], raising the possibility that the circadian clock has evolved to coordinate cell division with cellular metabolism, thus minimizing DNA damage. Furthermore, the NADH/NAD+ ratio and heme, indicators of the redox state, have been shown to directly modulate activity of circadian clock proteins, suggesting that the circadian clock can read and interpret the cellular metabolic state [56]. In addition, SIRT1, the mammalian orthologue of yeast SIR2, is a conserved NAD+-dependent protein deacetylase that deacetylates BMAL1 and PER2, and functions as a histone deacetylase at clock-regulated promoters [51,52,57]. SIR2 and its orthologues are important regulators of longevity in yeast, worms and flies [58-60], and in mice, several studies demonstrate SIRT1 contribution to genome stability and DNA repair [61,62].  Together, these data suggest the possibility that sirtuins could regulate longevity in part through circadian clock mechanisms [56].

Direct evidence for clock involvement in the aging process comes from the study of Kontdravov et. al.  [38], showing that the lifespan of Bmal1 mutant mice is decreased by half and that the mice exhibit a range of premature aging phenotypes. Among the phenotypes reported by the authors of this study were age-related lens and cornea defects, reduced subcutaneous fat, and hair regeneration defects, pointing to strong effect of the Bmal1 deletion in exposed cutaneous tissues, including hair follicles.  The authors demonstrate that by thirty weeks of age, Bmal1 deficient mice accumulate significantly more ROS than control animals, thus potentially explaining the progeria-like phenotype.  While our work focused on younger Bmal1 mutated mice [5], prior to the development of aging symptoms, the finding of circadian clock involvement in cell cycle progression within the secondary hair germ may provide a partial explanation for the hair regeneration defect. Alternatively, in the absence of BMAL1, deregulation of DNA damage and oxidative stress responses could cause depletion of stem populations necessary for hair regeneration.  Furthermore, in several rodent models there is deregulated suprachiasmatic nucleus electric activity and photic entrainment as well as abnormal periodicity and amplitude of circadian gene expression during the normal aging process [63-66].  Thus, a decline in the robustness of circadian rhythms may contribute to the aging process.

In conclusion, our study demonstrated that circadian clock genes can regulate the non-circadian cyclical hair growth cycle, presumably via an effect on the progression of the cell cycle in a progenitor cell compartment of the hair follicle, the secondary hair germ [5]. We speculate that circadian genes may play a role in aging-related alopecia which is characterized by aberrations in the hair growth cycle.
